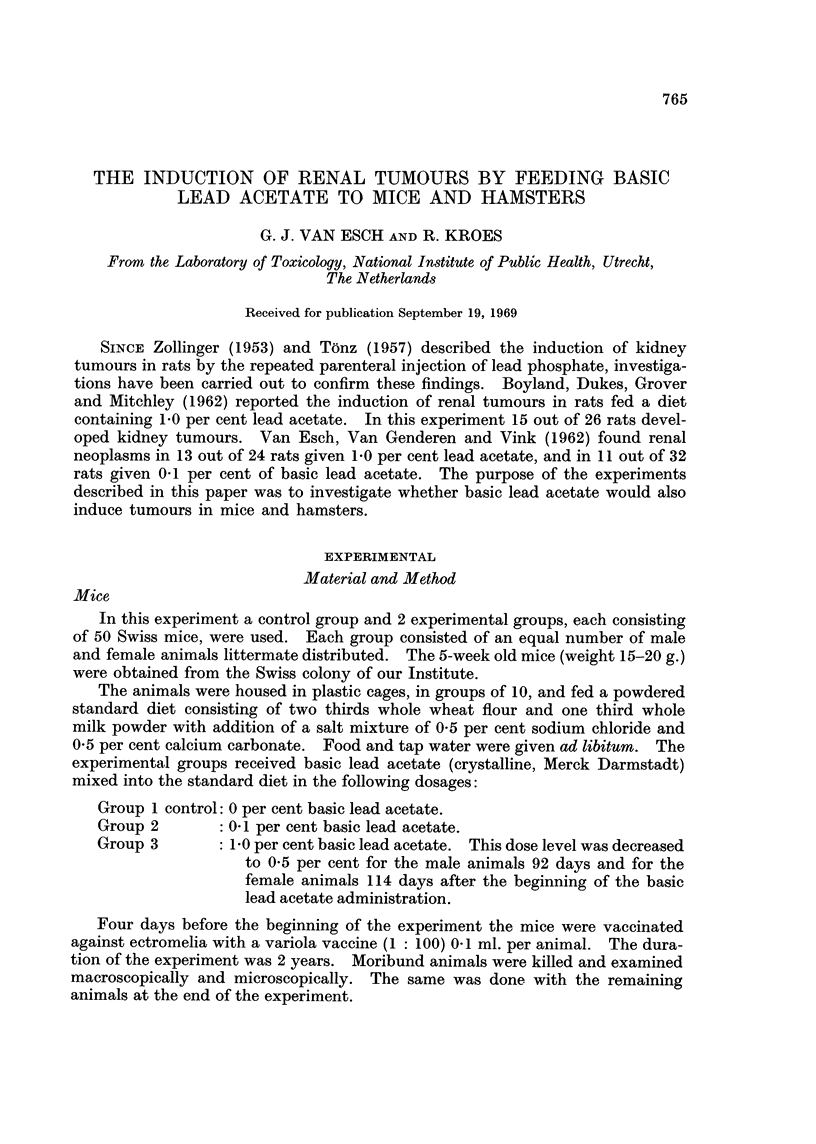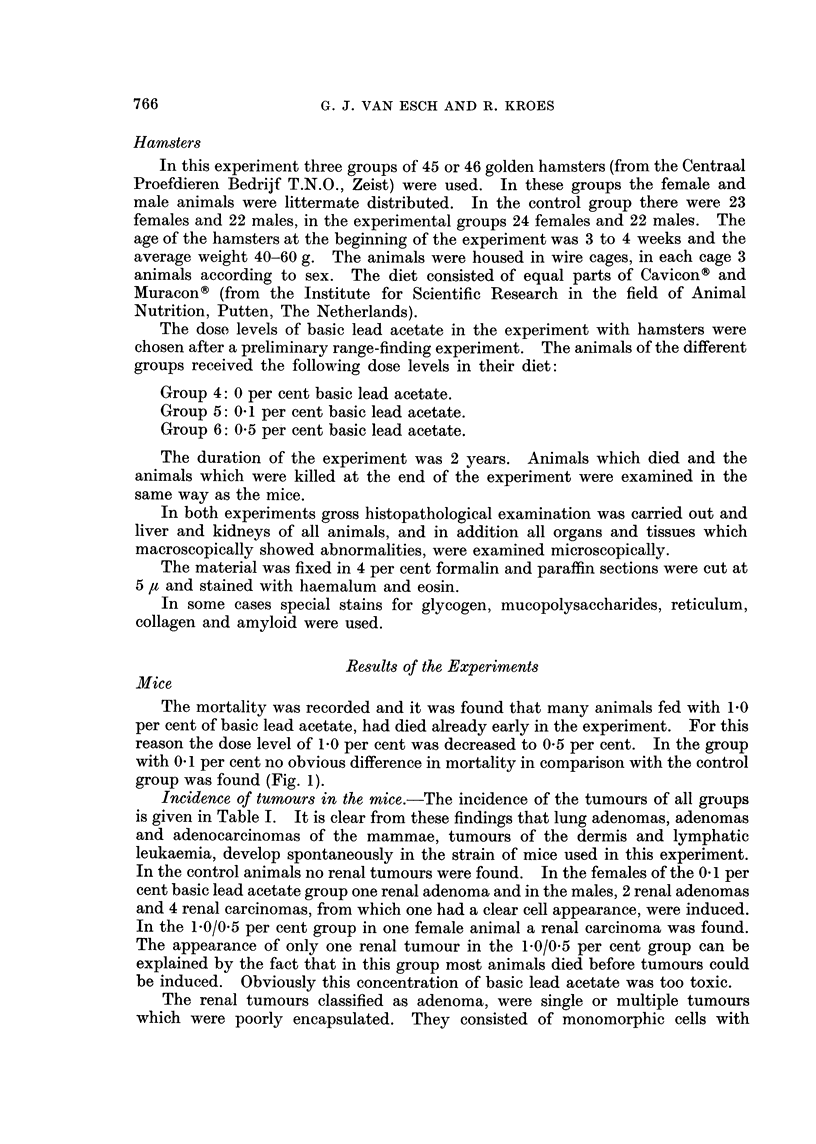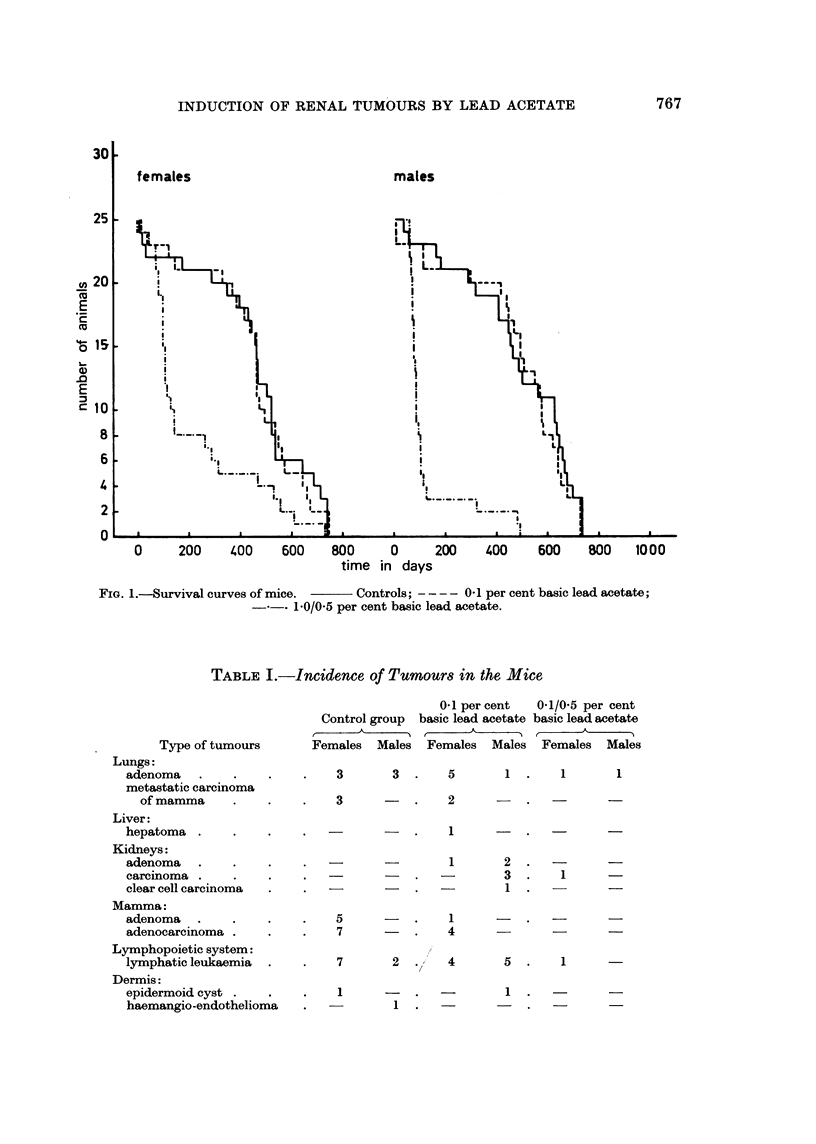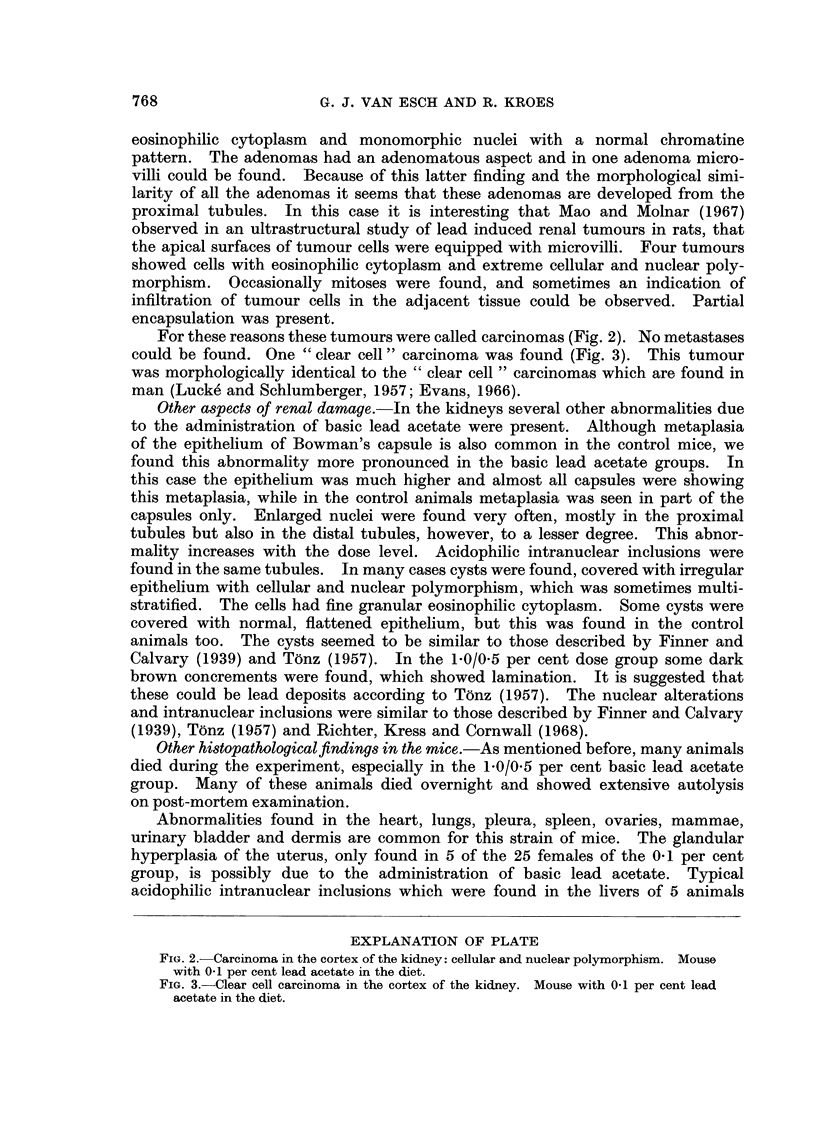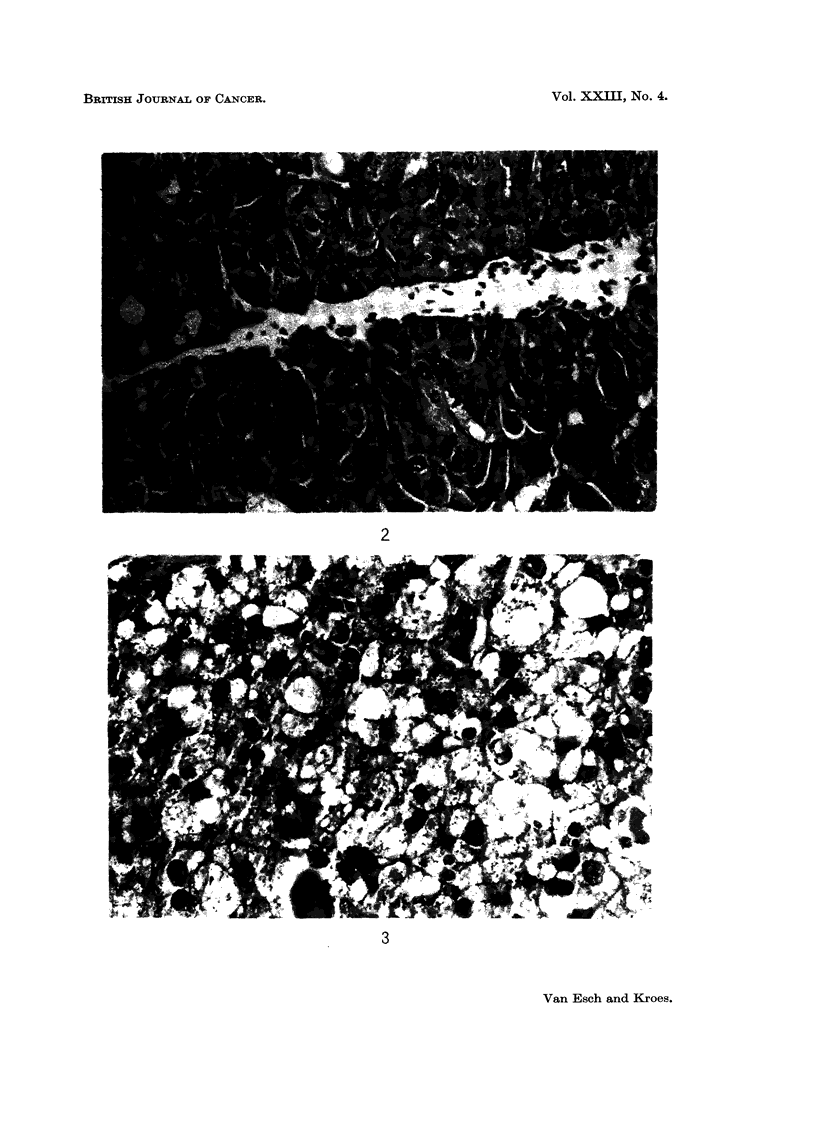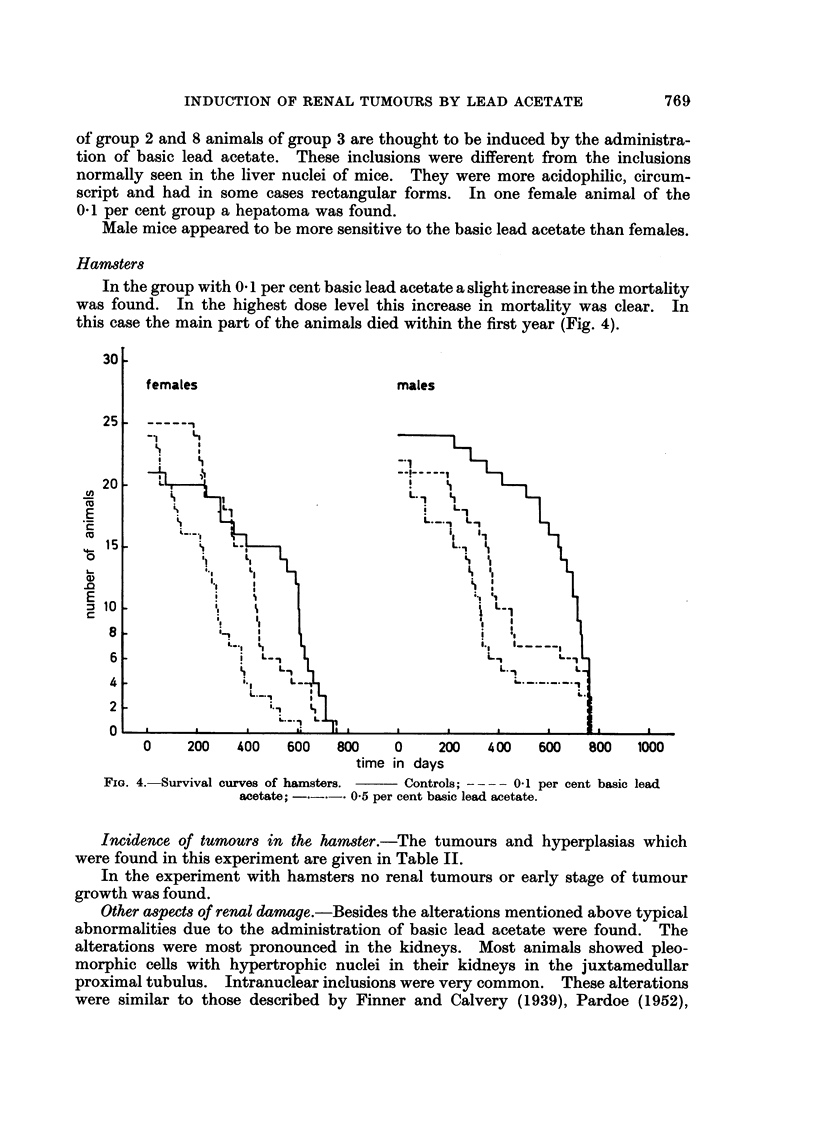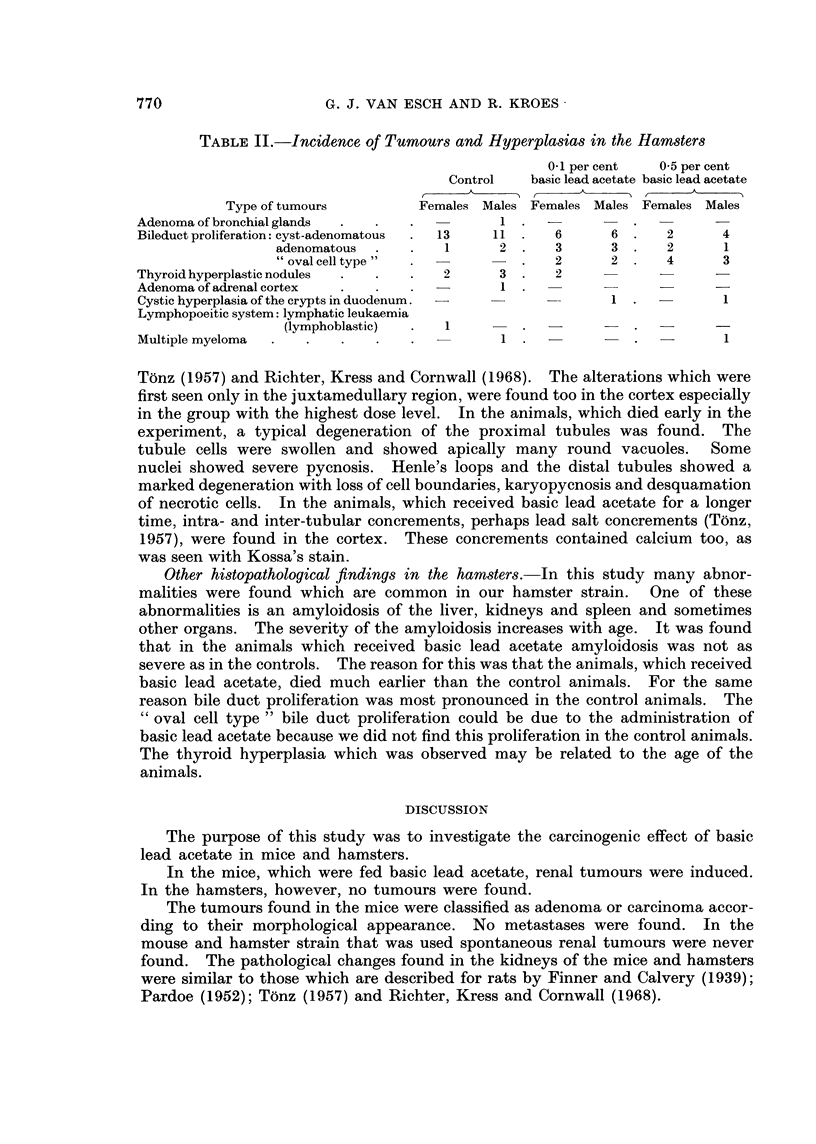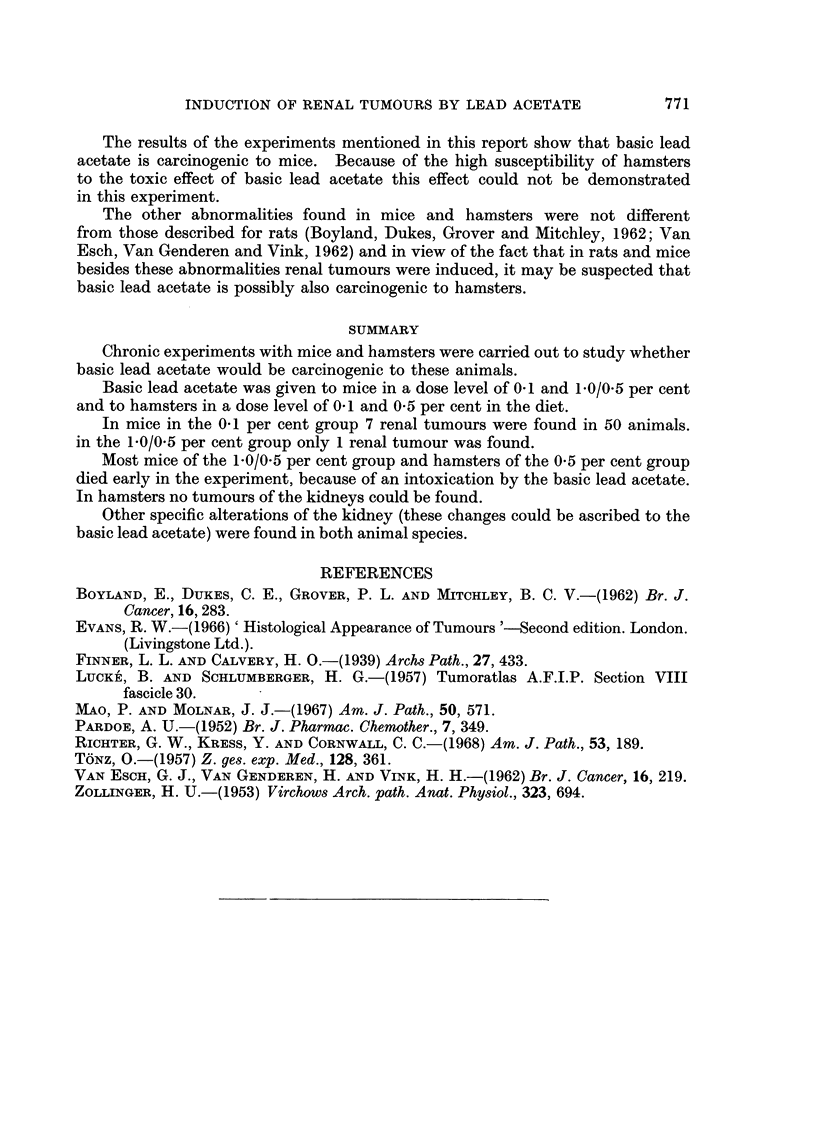# The induction of renal tumours by feeding basic lead acetate to mice and hamsters.

**DOI:** 10.1038/bjc.1969.95

**Published:** 1969-12

**Authors:** G. J. Van Esch, R. Kroes

## Abstract

**Images:**


### THE INDUCTION OF RENAL TUMOURS BY FEEDING BASIC LEAD ACETATE TO MICE AND HAMSTERS

G. J. VAN ESCH AND R. KROES

*From the Laboratory of Toxicology, National Institute of Public Health, Utrecht, The Netherlands*

Received for publication September 19, 1969

SINCE Zollinger (1953) and Tönz (1957) described the induction of kidney tumours in rats by the repeated parenteral injection of lead phosphate, investigations have been carried out to confirm these findings. Boyland, Dukes, Grover and Mitchley (1962) reported the induction of renal tumours in rats fed a diet containing 1·0 per cent lead acetate. In this experiment 15 out of 26 rats developed kidney tumours. Van Esch, Van Genderen and Vink (1962) found renal neoplasms in 13 out of 24 rats given 1·0 per cent lead acetate, and in 11 out of 32 rats given 0·1 per cent of basic lead acetate. The purpose of the experiments described in this paper was to investigate whether basic lead acetate would also induce tumours in mice and hamsters.

### EXPERIMENTAL

### *Material and Method*

*Mice*

In this experiment a control group and 2 experimental groups, each consisting of 50 Swiss mice, were used. Each group consisted of an equal number of male and female animals littermate distributed. The 5-week old mice (weight 15–20 g.) were obtained from the Swiss colony of our Institute.

The animals were housed in plastic cages, in groups of 10, and fed a powdered standard diet consisting of two thirds whole wheat flour and one third whole milk powder with addition of a salt mixture of 0·5 per cent sodium chloride and 0·5 per cent calcium carbonate. Food and tap water were given *ad libitum.* The experimental groups received basic lead acetate (crystalline, Merck Darmstadt) mixed into the standard diet in the following dosages:

Group 1 control: 0 per cent basic lead acetate.
Group 2 : 0·1 per cent basic lead acetate.
Group 3 : 1·0 per cent basic lead acetate. This dose level was decreased to 0·5 per cent for the male animals 92 days and for the female animals 114 days after the beginning of the basic lead acetate administration.

Four days before the beginning of the experiment the mice were vaccinated against ectromelia with a variola vaccine (1 : 100) 0·1 ml. per animal. The duration of the experiment was 2 years. Moribund animals were killed and examined macroscopically and microscopically. The same was done with the remaining animals at the end of the experiment.

*Hamsters*

In this experiment three groups of 45 or 46 golden hamsters (from the Centraal Proefdieren Bedrijf T.N.O., Zeist) were used. In these groups the female and male animals were littermate distributed. In the control group there were 23 females and 22 males, in the experimental groups 24 females and 22 males. The age of the hamsters at the beginning of the experiment was 3 to 4 weeks and the average weight 40–60 g. The animals were housed in wire cages, in each cage 3 animals according to sex. The diet consisted of equal parts of Cavicon® and Muracon® (from the Institute for Scientific Research in the field of Animal Nutrition, Putten, The Netherlands).

The dose levels of basic lead acetate in the experiment with hamsters were chosen after a preliminary range-finding experiment. The animals of the different groups received the following dose levels in their diet:

Group 4: 0 per cent basic lead acetate.
Group 5: 0·1 per cent basic lead acetate.
Group 6: 0·5 per cent basic lead acetate.

The duration of the experiment was 2 years. Animals which died and the animals which were killed at the end of the experiment were examined in the same way as the mice.

In both experiments gross histopathological examination was carried out and liver and kidneys of all animals, and in addition all organs and tissues which macroscopically showed abnormalities, were examined microscopically.

The material was fixed in 4 per cent formalin and paraffin sections were cut at 5 $\mu$ and stained with haemalum and eosin.

In some cases special stains for glycogen, mucopolysaccharides, reticulum, collagen and amyloid were used.

### *Results of the Experiments*

*Mice*

The mortality was recorded and it was found that many animals fed with 1·0 per cent of basic lead acetate, had died already early in the experiment. For this reason the dose level of 1·0 per cent was decreased to 0·5 per cent. In the group with 0·1 per cent no obvious difference in mortality in comparison with the control group was found (Fig. 1).

*Incidence of tumours in the mice.*—The incidence of the tumours of all groups is given in Table I. It is clear from these findings that lung adenomas, adenomas and adenocarcinomas of the mammae, tumours of the dermis and lymphatic leukaemia, develop spontaneously in the strain of mice used in this experiment. In the control animals no renal tumours were found. In the females of the 0·1 per cent basic lead acetate group one renal adenoma and in the males, 2 renal adenomas and 4 renal carcinomas, from which one had a clear cell appearance, were induced. In the 1·0/0·5 per cent group in one female animal a renal carcinoma was found. The appearance of only one renal tumour in the 1·0/0·5 per cent group can be explained by the fact that in this group most animals died before tumours could be induced. Obviously this concentration of basic lead acetate was too toxic.

The renal tumours classified as adenoma, were single or multiple tumours which were poorly encapsulated. They consisted of monomorphic cells with

FIG. 1.—Survival curves of mice. ——— Controls; – – – – 0·1 per cent basic lead acetate; —·— 1·0/0·5 per cent basic lead acetate.

TABLE I.—*Incidence of Tumours in the Mice*

| Type of tumours | Control group | | 0·1 per cent basic lead acetate | | 0·1/0·5 per cent basic lead acetate | |
|---|---|---|---|---|---|---|
| | Females | Males | Females | Males | Females | Males |
| Lungs: | | | | | | |
| adenoma | 3 | 3 | 5 | 1 | 1 | 1 |
| metastatic carcinoma of mamma | 3 | — | 2 | — | — | — |
| Liver: | | | | | | |
| hepatoma | — | — | 1 | — | — | — |
| Kidneys: | | | | | | |
| adenoma | — | — | 1 | 2 | — | — |
| carcinoma | — | — | — | 3 | 1 | — |
| clear cell carcinoma | — | — | — | 1 | — | — |
| Mamma: | | | | | | |
| adenoma | 5 | — | 1 | — | — | — |
| adenocarcinoma | 7 | — | 4 | — | — | — |
| Lymphopoietic system: | | | | | | |
| lymphatic leukaemia | 7 | 2 | 4 | 5 | 1 | — |
| Dermis: | | | | | | |
| epidermoid cyst | 1 | — | — | 1 | — | — |
| haemangio-endothelioma | — | 1 | — | — | — | — |

eosinophilic cytoplasm and monomorphic nuclei with a normal chromatine pattern. The adenomas had an adenomatous aspect and in one adenoma microvilli could be found. Because of this latter finding and the morphological similarity of all the adenomas it seems that these adenomas are developed from the proximal tubules. In this case it is interesting that Mao and Molnar (1967) observed in an ultrastructural study of lead induced renal tumours in rats, that the apical surfaces of tumour cells were equipped with microvilli. Four tumours showed cells with eosinophilic cytoplasm and extreme cellular and nuclear polymorphism. Occasionally mitoses were found, and sometimes an indication of infiltration of tumour cells in the adjacent tissue could be observed. Partial encapsulation was present.

For these reasons these tumours were called carcinomas (Fig. 2). No metastases could be found. One "clear cell" carcinoma was found (Fig. 3). This tumour was morphologically identical to the "clear cell" carcinomas which are found in man (Lucké and Schlumberger, 1957; Evans, 1966).

*Other aspects of renal damage.*—In the kidneys several other abnormalities due to the administration of basic lead acetate were present. Although metaplasia of the epithelium of Bowman's capsule is also common in the control mice, we found this abnormality more pronounced in the basic lead acetate groups. In this case the epithelium was much higher and almost all capsules were showing this metaplasia, while in the control animals metaplasia was seen in part of the capsules only. Enlarged nuclei were found very often, mostly in the proximal tubules but also in the distal tubules, however, to a lesser degree. This abnormality increases with the dose level. Acidophilic intranuclear inclusions were found in the same tubules. In many cases cysts were found, covered with irregular epithelium with cellular and nuclear polymorphism, which was sometimes multistratified. The cells had fine granular eosinophilic cytoplasm. Some cysts were covered with normal, flattened epithelium, but this was found in the control animals too. The cysts seemed to be similar to those described by Finner and Calvary (1939) and Tönz (1957). In the 1·0/0·5 per cent dose group some dark brown concrements were found, which showed lamination. It is suggested that these could be lead deposits according to Tönz (1957). The nuclear alterations and intranuclear inclusions were similar to those described by Finner and Calvary (1939), Tönz (1957) and Richter, Kress and Cornwall (1968).

*Other histopathological findings in the mice.*—As mentioned before, many animals died during the experiment, especially in the 1·0/0·5 per cent basic lead acetate group. Many of these animals died overnight and showed extensive autolysis on post-mortem examination.

Abnormalities found in the heart, lungs, pleura, spleen, ovaries, mammae, urinary bladder and dermis are common for this strain of mice. The glandular hyperplasia of the uterus, only found in 5 of the 25 females of the 0·1 per cent group, is possibly due to the administration of basic lead acetate. Typical acidophilic intranuclear inclusions which were found in the livers of 5 animals

---

EXPLANATION OF PLATE

FIG. 2.—Carcinoma in the cortex of the kidney: cellular and nuclear polymorphism. Mouse with 0·1 per cent lead acetate in the diet.

FIG. 3.—Clear cell carcinoma in the cortex of the kidney. Mouse with 0·1 per cent lead acetate in the diet.

2

3

Van Esch and Kroes.

of group 2 and 8 animals of group 3 are thought to be induced by the administration of basic lead acetate. These inclusions were different from the inclusions normally seen in the liver nuclei of mice. They were more acidophilic, circumscript and had in some cases rectangular forms. In one female animal of the 0·1 per cent group a hepatoma was found.

Male mice appeared to be more sensitive to the basic lead acetate than females.

*Hamsters*

In the group with 0·1 per cent basic lead acetate a slight increase in the mortality was found. In the highest dose level this increase in mortality was clear. In this case the main part of the animals died within the first year (Fig. 4).

FIG. 4.—Survival curves of hamsters. ——— Controls; - - - - 0·1 per cent basic lead acetate; —·—·— 0·5 per cent basic lead acetate.

*Incidence of tumours in the hamster.*—The tumours and hyperplasias which were found in this experiment are given in Table II.

In the experiment with hamsters no renal tumours or early stage of tumour growth was found.

*Other aspects of renal damage.*—Besides the alterations mentioned above typical abnormalities due to the administration of basic lead acetate were found. The alterations were most pronounced in the kidneys. Most animals showed pleomorphic cells with hypertrophic nuclei in their kidneys in the juxtamedullar proximal tubulus. Intranuclear inclusions were very common. These alterations were similar to those described by Finner and Calvery (1939), Pardoe (1952),

TABLE II.—*Incidence of Tumours and Hyperplasias in the Hamsters*

| Type of tumours | Control Females | Control Males | 0·1 per cent basic lead acetate Females | 0·1 per cent basic lead acetate Males | 0·5 per cent basic lead acetate Females | 0·5 per cent basic lead acetate Males |
|---|---|---|---|---|---|---|
| Adenoma of bronchial glands | — | 1 | — | — | — | — |
| Bileduct proliferation: cyst-adenomatous | 13 | 11 | 6 | 6 | 2 | 4 |
| adenomatous | 1 | 2 | 3 | 3 | 2 | 1 |
| " oval cell type " | — | — | 2 | 2 | 4 | 3 |
| Thyroid hyperplastic nodules | 2 | 3 | 2 | — | — | — |
| Adenoma of adrenal cortex | — | 1 | — | — | — | — |
| Cystic hyperplasia of the crypts in duodenum | — | — | — | 1 | — | 1 |
| Lymphopoeitic system: lymphatic leukaemia (lymphoblastic) | 1 | — | — | — | — | — |
| Multiple myeloma | — | 1 | — | — | — | 1 |

Tönz (1957) and Richter, Kress and Cornwall (1968). The alterations which were first seen only in the juxtamedullary region, were found too in the cortex especially in the group with the highest dose level. In the animals, which died early in the experiment, a typical degeneration of the proximal tubules was found. The tubule cells were swollen and showed apically many round vacuoles. Some nuclei showed severe pycnosis. Henle's loops and the distal tubules showed a marked degeneration with loss of cell boundaries, karyopycnosis and desquamation of necrotic cells. In the animals, which received basic lead acetate for a longer time, intra- and inter-tubular concrements, perhaps lead salt concrements (Tönz, 1957), were found in the cortex. These concrements contained calcium too, as was seen with Kossa's stain.

*Other histopathological findings in the hamsters.*—In this study many abnormalities were found which are common in our hamster strain. One of these abnormalities is an amyloidosis of the liver, kidneys and spleen and sometimes other organs. The severity of the amyloidosis increases with age. It was found that in the animals which received basic lead acetate amyloidosis was not as severe as in the controls. The reason for this was that the animals, which received basic lead acetate, died much earlier than the control animals. For the same reason bile duct proliferation was most pronounced in the control animals. The " oval cell type " bile duct proliferation could be due to the administration of basic lead acetate because we did not find this proliferation in the control animals. The thyroid hyperplasia which was observed may be related to the age of the animals.

### DISCUSSION

The purpose of this study was to investigate the carcinogenic effect of basic lead acetate in mice and hamsters.

In the mice, which were fed basic lead acetate, renal tumours were induced. In the hamsters, however, no tumours were found.

The tumours found in the mice were classified as adenoma or carcinoma according to their morphological appearance. No metastases were found. In the mouse and hamster strain that was used spontaneous renal tumours were never found. The pathological changes found in the kidneys of the mice and hamsters were similar to those which are described for rats by Finner and Calvery (1939); Pardoe (1952); Tönz (1957) and Richter, Kress and Cornwall (1968).

The results of the experiments mentioned in this report show that basic lead acetate is carcinogenic to mice. Because of the high susceptibility of hamsters to the toxic effect of basic lead acetate this effect could not be demonstrated in this experiment.

The other abnormalities found in mice and hamsters were not different from those described for rats (Boyland, Dukes, Grover and Mitchley, 1962; Van Esch, Van Genderen and Vink, 1962) and in view of the fact that in rats and mice besides these abnormalities renal tumours were induced, it may be suspected that basic lead acetate is possibly also carcinogenic to hamsters.

### SUMMARY

Chronic experiments with mice and hamsters were carried out to study whether basic lead acetate would be carcinogenic to these animals.

Basic lead acetate was given to mice in a dose level of 0·1 and 1·0/0·5 per cent and to hamsters in a dose level of 0·1 and 0·5 per cent in the diet.

In mice in the 0·1 per cent group 7 renal tumours were found in 50 animals. in the 1·0/0·5 per cent group only 1 renal tumour was found.

Most mice of the 1·0/0·5 per cent group and hamsters of the 0·5 per cent group died early in the experiment, because of an intoxication by the basic lead acetate. In hamsters no tumours of the kidneys could be found.

Other specific alterations of the kidney (these changes could be ascribed to the basic lead acetate) were found in both animal species.

### REFERENCES

Boyland, E., Dukes, C. E., Grover, P. L. and Mitchley, B. C. V.—(1962) *Br. J. Cancer*, **16**, 283.

Evans, R. W.—(1966) ‘ Histological Appearance of Tumours ’—Second edition. London. (Livingstone Ltd.).

Finner, L. L. and Calvery, H. O.—(1939) *Archs Path.*, **27**, 433.

Lucké, B. and Schlumberger, H. G.—(1957) Tumoratlas A.F.I.P. Section VIII fascicle 30.

Mao, P. and Molnar, J. J.—(1967) *Am. J. Path.*, **50**, 571.

Pardoe, A. U.—(1952) *Br. J. Pharmac. Chemother.*, **7**, 349.

Richter, G. W., Kress, Y. and Cornwall, C. C.—(1968) *Am. J. Path.*, **53**, 189.

Tönz, O.—(1957) *Z. ges. exp. Med.*, **128**, 361.

Van Esch, G. J., Van Genderen, H. and Vink, H. H.—(1962) *Br. J. Cancer*, **16**, 219.

Zollinger, H. U.—(1953) *Virchows Arch. path. Anat. Physiol.*, **323**, 694.